# Takeda G protein–coupled receptor 5 (TGR5): an attractive therapeutic target for aging-related cardiovascular diseases

**DOI:** 10.3389/fphar.2025.1493662

**Published:** 2025-03-20

**Authors:** Yufeng He, Siqi Liu, Yali Zhang, Yumei Zuo, Keming Huang, Li Deng, Bin Liao, Yi Zhong, Jian Feng

**Affiliations:** ^1^ Department of Cardiology, The Affiliated Hospital of Southwest Medical University, Luzhou, Sichuan, China; ^2^ Department of Rheumatology, The Affiliated Hospital of Southwest Medical University, Luzhou, Sichuan, China; ^3^ Metabolic Vascular Diseases Key Laboratory of Sichuan Province, Department of Cardiovascular Surgery, The Affiliated Hospital of Southwest Medical University, Luzhou, Sichuan, China

**Keywords:** aging, cardiovascular disease, TGR5, heart failure, myocardial infarction

## Abstract

Aging is an independent risk factor for many chronic diseases, including cancer and cardiovascular, pulmonary, and neurodegenerative diseases. In recent years, the mechanisms of aging-related cardiovascular diseases (CVDs) have been studied intensively. Takeda G protein-coupled receptor 5 (TGR5) is a membrane receptor for bile acids that has been found to play an important role in various disease processes, such as inflammation, oxidative stress, and metabolic disorders, all of which contribute to aging-related CVDs. In this review, we summarise the role of TGR5 in aging-related CVDs and propose TGR5 as an attractive therapeutic target based on its mechanism of involvement, which may contribute to future drug target design.

## 1 Introduction

With the progressive aging of the global population, the incidence of aging-related CVDs has been on the rise (Sheikh and Salma, 2024). Despite much work has been done to ameliorate or prevent age-related physiological decline and to select appropriate therapeutic agents to maximize healthy lifespan, effective clinical interventions to mitigate the impact of aging on human health remain limited (Queen et al., 2020; Hodgson et al., 2020; Lacanna et al., 2019; Ali et al., 2020). Therefore, it is imperative that we explore therapeutic approaches to promote healthy aging.

One promising therapeutic target that has gained attention in the context of aging is Takeda G protein-coupled receptor 5 (TGR5). It plays a crucial role in the regulation of metabolic processes such as glucose and lipid metabolism, as well as modulating immune-inflammatory responses and maintaining energy homeostasis (Jin et al., 2024). According to previous researches, TGR5 has been reported to have a significant impact on the progression of tumors and the development of metabolic disorders such as diabetes and obesity (Jin et al., 2024; Lun et al., 2024; Zhao et al., 2022; Bhimanwar and Mittal, 2022). More recently, emerging evidence has suggested that TGR5 may also play a pivotal role in the aging process (Wang et al., 2023; Perino et al., 2021). In this review, we aim to summarise the role of TGR5 in aging-related cardiovascular diseases and to explore the therapeutic potential of TGR5 for targeting these diseases. By understanding the intricate interplay between TGR5 and the various pathways involved in cardiovascular aging, we hope to pave the way for the development of therapeutic strategies to combat aging-related CVDs.

## 2 Fundamental functions and distribution of TGR5

TGR5, a member of the G protein-coupled receptor (GPCR) superfamily, is characterized by seven transmembrane domains that enable it to recognize and bind various bile acid molecules, including deoxycholic acid (DCA) and lithocholic acid (LCA) (Lun et al., 2024). Upon activation, TGR5 primarily increases intracellular cAMP levels through the stimulation of Gs protein (Tanaka et al., 2024). As a stimulatory G protein, Gs interacts with the activated TGR5, leading to the activation of adenylate cyclase (AC). This subsequently promotes the conversion of ATP into cAMP. This process constitutes a crucial step in the TGR5 signaling pathway, as cAMP functions as a second messenger to further activate downstream signaling molecules, including protein kinase A (PKA), thereby regulating a wide range of cellular physiological functions (Holter et al., 2020). These signaling events subsequently exert influences on the physiological functions of cells.

TGR5 is widely expressed across various tissues and organs, reflecting its diverse physiological roles. High levels of TGR5 expression have been observed in the liver, intestine, kidneys, and cardiovascular system (Gou et al., 2023). In the liver, TGR5 is expressed on the surface of hepatocytes and cholangiocytes, playing a pivotal role in bile acid homeostasis and liver protection (Bidault-Jourdainne et al., 2021). By sensing changes in bile acid levels, TGR5 regulates bile acid synthesis and transport, thereby preventing the accumulation of toxic bile acids and protecting the liver from injury (Chiang and Ferrell, 2020). In the intestine, TGR5 is expressed on the surface of enterocytes, where it promotes the absorption of bile acids and fatty acids and regulates intestinal motility (Chang et al., 2024). Through facilitating nutrient absorption and modulating gut function, TGR5 contributes to overall metabolic health. In the kidneys, TGR5 is expressed on the surface of renal tubular cells (Guo et al., 2024). By modulating renal function, TGR5 helps prevent the progression of kidney diseases, such as nephropathy (Kim et al., 2023). Finally, in the cardiovascular system, TGR5 is expressed on cardiomyocytes, endothelial cells, and smooth muscle cells, where it regulates cardiac function, vascular tone, and inflammation (Wang et al., 2021; Sauerbruch et al., 2021).

## 3 TGR5 in aging-related CVDs

### 3.1 Atherosclerosis

Atherosclerosis is a typical aging-related chronic CVD that poses a significant threat to human health and is one of the leading causes of death among the elderly (Kong et al., 2022). As individuals age, metabolic and immune functions decline, increasing the risk of atherosclerosis. Research has shown that TGR5 expression levels significantly decrease in aging mouse models, whereas TGR5 activation significantly improves aging-related cardiovascular dysfunction (Wang et al., 2017). In animal models, TGR5 activation has significantly reduced plasma total cholesterol and triglyceride levels, as well as atherosclerotic lesion areas in the aortic root, underscoring its crucial role in regulating lipid metabolism (Biagioli et al., 2023). Additionally, in macrophages, TGR5 activation downregulates the expression of scavenger receptor A (SR-A) and cluster of differentiation 36 (CD36), which are pivotal in the uptake of oxidized LDL (oxLDL) and foam cell formation (Xia et al., 2020). Consequently, TGR5 activation reduces the uptake of oxLDL by macrophages, inhibiting foam cell formation and mitigating the lipid deposition process in atherosclerosis. These suggest that TGR5 plays a pivotal role in regulating various physiological and pathophysiological processes, exhibiting remarkable protective effects against the development of atherosclerosis.

**FIGURE 1:**
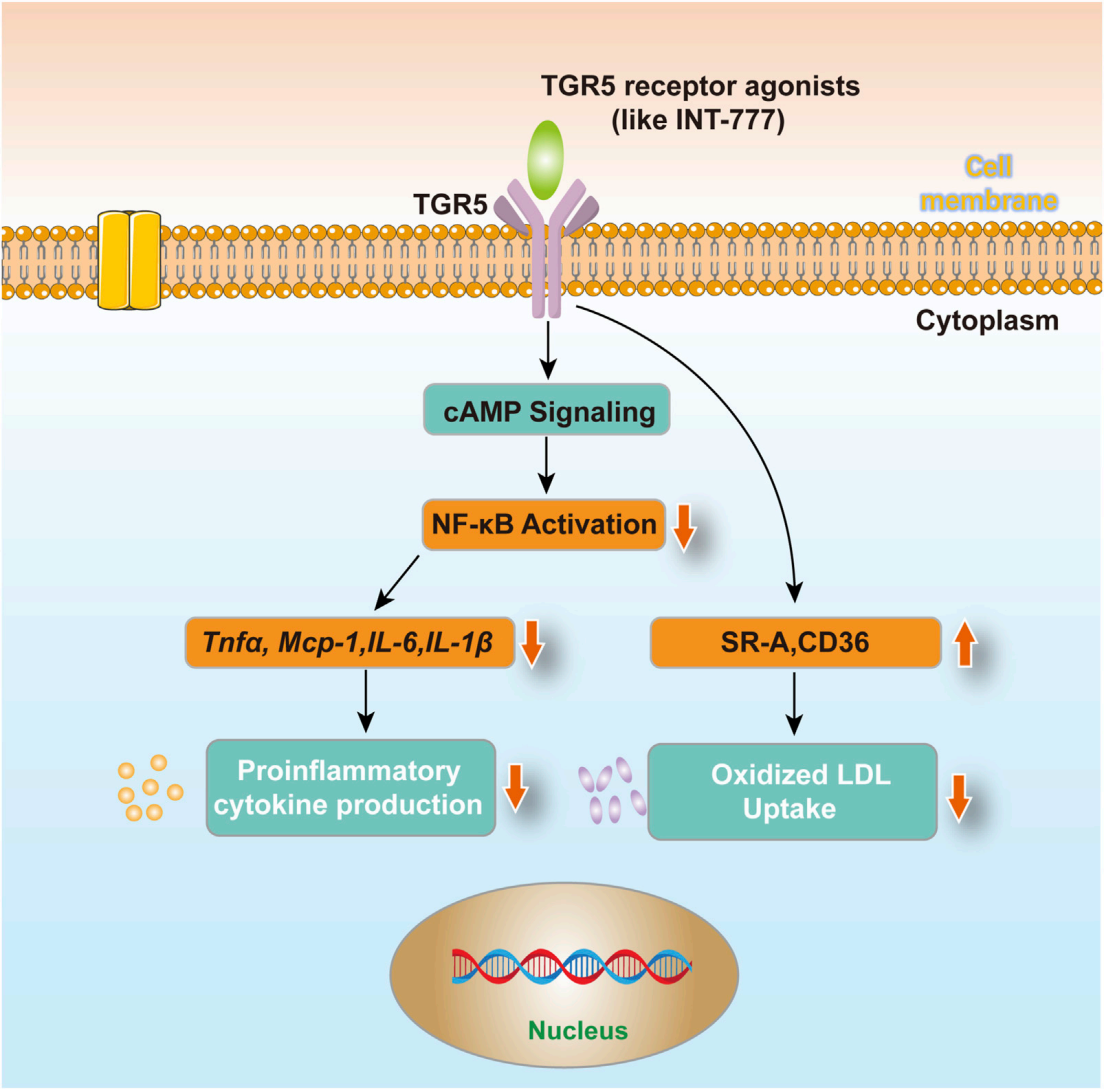
TGR5 in atherosclerosis. After using TGR5 receptor agonists (such as INT-777) treated cells, macrophages reduce the production of circulating factors and uptake of oxidized LDL via TGR5-induced cAMP signaling pathway and subsequent NF-κB inhibition, which may contribute to its anti-atherosclerotic effect.

Given TGR5’s multifaceted protective roles in atherosclerosis, its potential as a therapeutic target is gaining increasing attention. Various TGR5 agonists have been developed and demonstrated promising anti-atherosclerotic effects in animal models. For instance, INT-777 significantly inhibits the development of atherosclerosis in mouse models, reducing macrophage content within plaques (Miyazaki-Anzai et al., 2018). Additionally, the application of nanotechnology has enhanced the bioavailability and targeting of TGR5 agonists, laying the groundwork for future clinical trials (Moretti et al., 2018). Figure 1 shows the role of TGR5 in atherosclerosis. However, therapeutic strategies targeting TGR5 still face significant challenges. For instance, systemic activation of TGR5 agonists may disrupt bile acid homeostasis, potentially inducing biliary dysfunction or gastrointestinal adverse effects (Fleishman and Kumar, 2024; Ye et al., 2024). Additionally, the extent of TGR5 signaling activation may vary due to individual genetic backgrounds (such as GPBAR1 polymorphisms) or differences in gut microbiota composition, leading to inconsistent therapeutic responses (Zhao et al., 2023; Pavlovic et al., 2017). The dose-dependent effects observed in preclinical studies also suggest that careful optimization of dosing regimens in human trials is necessary to avoid desensitization or receptor downregulation risks (Van Nierop et al., 2017).

### 3.2 Myocardial infarction (MI) and heart failure (HF)

MI is predominantly observed in the elders. However, dysregulation of immune pathways, impaired suppression of post-MI inflammation, spatially restricted inflammatory responses, and excessive fibrosis can lead to adverse cardiac remodeling, significantly impairing cardiac function. In recent years, the role of bile acids and their receptors in CVDs has garnered increasing attention. A study investigating changes in plasma bile acid levels in patients with acute MI found significantly reduced levels of DCA (Wang et al., 2021). This observation led researchers to investigate the role of DCA and its receptor TGR5 in the context of MI. In a mouse model of MI induced by ligation of the left anterior descending coronary artery, subsequent studies demonstrated that DCA binding to TGR5 significantly improved cardiac function and reduced ischemic injury (Wang et al., 2021). These findings highlight the protective role of DCA in MI and suggest that TGR5 could be a promising therapeutic target. Additionally, DCA was found to suppress the expression and activation of IL-1β, thereby mitigating inflammation-induced myocardial damage (Forteza et al., 2023).

To further explore the role of TGR5 in MI, researchers have conducted investigations into its impact on the immune system. In TGR5^−/−^ mice, the proportions of CD4^+^ T and CD8^+^ T cells in the heart and spleen remained elevated, with significantly increased CD4^+^ T cell infiltration in ischemic areas (Cheng et al., 2022). This suggests that TGR5 may regulate CD4^+^ T cell function post-MI. To test this hypothesis, researchers performed MI surgeries in CD4-specific TGR5-knockout mice. Compared to controls, these mice exhibited more severe ischemic injury, significantly reduced cardiac function, and larger infarct areas, further confirming TGR5’s critical role in post-MI cardiac repair and remodeling (Cheng et al., 2022).

At the cellular level, additional studies have shed light on TGR5’s mechanisms of action. In rat H9C2 cardiomyocytes and human cardiomyocytes subjected to ischemia/reperfusion injury, TGR5 expression was significantly upregulated (Li et al., 2020). This upregulation is likely an adaptive response to ischemic injury, aiming to mitigate damage through TGR5 activation. Further experiments revealed that TGR5 activation improved cell proliferation, reduced apoptosis, and decreased the mRNA and protein levels of inflammatory factors, suggesting that TGR5 confers cardioprotective effects by suppressing inflammation and enhancing cell survival (Li et al., 2020).

Notably, the pro-survival effects of TGR5 activation may entail metabolic trade-offs (Su et al., 2024; Guo et al., 2016). Enhanced cAMP signaling could exacerbate arrhythmogenic risks, particularly in aged hearts with preexisting calcium dysregulation (Grammatika Pavlidou et al., 2023; Lin et al., 2018). Interestingly, the protective effects of TGR5 were partially reversed when cells were treated with the AKT inhibitor MK-2206 (Li et al., 2020). This finding indicates that TGR5 may exert its protective functions through the AKT/GSK-3β signaling pathway. Given the pivotal role of the AKT/GSK-3β pathway in cell survival, proliferation, and apoptosis, TGR5 likely modulates this pathway to safeguard cardiomyocytes from ischemic injury. Additionally, the dual role of TGR5 in post-ischemic inflammation resolution and fibrotic repair remains incompletely understood, necessitating time-dependent modulation to avoid maladaptive remodeling (Wang et al., 2022; Kaya et al., 2019).

HF is a terminal stage of various CVDs, is characterized by diminished cardiac pump function. Recent studies have revealed a close relationship between TGR5 and aging-related HF. Changes in TGR5 expression and function in elderly HF patients may influence cardiac function and repair processes (Xu et al., 2024). MI is a major trigger for HF, as myocardial necrosis and scar formation after MI lead to structural and functional alterations in the heart, predisposing it to HF (Jiang et al., 2023). TGR5 likely plays a pivotal role in this transition by promoting myocardial repair and regeneration, reducing inflammation, and improving cardiac function, thereby preventing HF progression (Yan et al., 2024).

### 3.3 Cardiac hypertrophy

In the elderly population, cardiac hypertrophy is often pathological and closely related to cardiac dysfunction, significantly impacting their quality of life and overall health status (Tracy et al., 2020). Multiple studies have confirmed that the activation of TGR5 can inhibit the onset and progression of cardiac hypertrophy. In an H9C2 cardiac hypertrophy model induced by high glucose, LCA reduces the mRNA levels of hypertrophic markers such as calcineurin (CaN) and nuclear factor of activated T-cells (NFAT) in a dose-dependent manner (Cheng et al., 2019). Furthermore, upon activation of TGR5 by LCA, cAMP levels rise, subsequently activating PKA and increasing the phosphorylation of phospholamban (PLN) (Cheng et al., 2019). This process reverses the downregulation of SERCA2a expression, which is a crucial step in improving cardiac function. These findings suggest that TGR5 activation modulates intracellular calcium homeostasis and inhibits the CaN/NFAT signaling pathway, thereby reducing calcium ion accumulation in cardiomyocytes and promoting myocardial energy metabolism, ultimately inhibiting cardiomyocyte hypertrophy.

Additionally, in a myocardial hypertrophy model induced by endothelin-1 (ET-1), TGR5 activation significantly suppressed the increase in cardiomyocyte surface area and total protein, as well as the expression of hypertrophic markers such as atrial natriuretic peptide (ANF) and β-myosin heavy chain (β-MHC) (Chi et al., 2024; Jen et al., 2017; Wang et al., 2024). TGR5 activation may also protect cardiomyocytes from damage by inhibiting inflammation and oxidative stress, further inhibiting cardiac hypertrophy (Xu et al., 2024; Cheng et al., 2019).

In a DCM model, TGR5 plays a regulatory role in cardiac hypertrophy. Cardiac-specific TGR5^−/−^ mice exhibit more severe cardiac dysfunction and myocardial hypertrophy, while TGR5 activation can prevent lipotoxicity and cardiac dysfunction, inhibiting cardiac hypertrophy (Fleishman and Kumar, 2024; Wang et al., 2024). This may be related to TGR5’s inhibition of fatty acid uptake and the localization of CD36 in the plasma membrane, as CD36 is the main mediator of long-chain fatty acid uptake. By inhibiting CD36-mediated fatty acid uptake and lipotoxicity, the energy metabolism and inflammatory responses of cardiac cells are improved, thereby slowing or reversing the development of cardiac hypertrophy (Shu et al., 2022).

### 3.4 Cardiac fibrosis

During the aging process, the risk of cardiac fibrosis significantly increases (Izzo et al., 2021). Cardiac fibrosis serves as a common pathological basis for various CVDs, including HF and MI, and is also a critical factor contributing to the gradual decline of cardiac function (Jiang et al., 2021).

Emerging evidence suggests that the activation of TGR5 may inhibit the onset and progression of cardiac fibrosis through multiple pathways. With advancing age, the body’s energy metabolism and lipid metabolism functions gradually decline, resulting in abnormal lipid deposition in tissues such as the heart (Xie et al., 2023). However, the activation of TGR5 can promote energy expenditure and lipid metabolism, reducing lipid accumulation in cardiac tissue and thereby effectively inhibiting the occurrence of cardiac fibrosis (Wang et al., 2024). The activation of TGR5, on the other hand, can protect cardiac tissue from injury by inhibiting inflammatory responses and oxidative stress pathways (Doroszko et al., 2020). In animal models, it has been observed that TGR5 activation reduces the expression of inflammatory factors and the generation of oxidative stress products in myocardial tissue, thereby inhibiting the progression of cardiac fibrosis (Xu et al., 2024; Yin et al., 2023). The activation of TGR5 may regulate the expression of genes related to extracellular matrix (ECM) synthesis and degradation, suppressing the activation and proliferation of cardiac fibroblasts, and thus reducing collagen fiber deposition (Yin et al., 2023). For example, by inhibiting the expression of fibrosis factors such as transforming growth factor-β (TGF-β), TGR5 can decrease the activation of cardiac fibroblasts and the synthesis of ECM (Reilly-O'Donnell et al., 2024; Sweeney et al., 2020). In a mouse model of myocardial fibrosis induced by a combination of isoproterenol and ursodeoxycholic acid (UDCA), research has found that UDCA, through the activation of TGR5, significantly alleviated the degree of myocardial fibrosis and reduced the expression of type I and III collagen, as well as TGF-β1 protein (Reilly-O'Donnell et al., 2024; Li et al., 2017). This finding further supports the potential role of TGR5 in cardiac fibrosis.

### 3.5 Hypertension

Hypertension, as a chronic disease that is widespread globally, has a significantly higher prevalence with age, posing a serious threat to public health (Mills et al., 2020). During the aging process, the activation of TGR5 stimulates energy expenditure and lipid metabolism, reducing the accumulation of lipids in vital tissues such as the heart and blood vessels, thereby lowering the risk of hypertension (Wang et al., 2023). Furthermore, TGR5 can inhibit the expression of the epithelial sodium channel (ENaC) in the kidney, decreasing sodium reabsorption and subsequently lowering blood pressure (Xu et al., 2025). This mechanism has been validated in mouse models treated with deoxycorticosterone acetate (DOCA), highlighting the significant role of TGR5 in renal sodium handling (Xu et al., 2025).

Beyond its direct effects on the kidney, TGR5 also participates in blood pressure regulation by influencing water and electrolyte balance. Studies have shown that TGR5 activation can modulate the expression and trafficking of AQP2 (aquaporin-2) in renal medullary collecting duct cells via the cAMP pathway, thereby affecting water reabsorption and urine concentration, and indirectly regulating blood pressure (Guo et al., 2024). Additionally, TGR5 activation induces the expression of eNOS and the production of NO, while promoting the generation of hydrogen sulfide and inhibiting endothelin signaling (Ma et al., 2021; Fiorucci et al., 2017). These effects collectively promote vasodilation and reduce blood pressure. Nevertheless, TGR5-mediated regulation of renal ENaC and AQP2 may be attenuated by age-related tubular dysfunction, contributing to interindividual variability in antihypertensive efficacy (Tamma et al., 2015; Tiwari et al., 2009). Moreover, central TGR5 activation, which suppresses sympathetic activity, may unpredictably interact with conventional therapies (such as β-blockers), warranting pharmacokinetic studies to elucidate synergistic or antagonistic mechanisms.

Notably, the activation of TGR5 can significantly ameliorate hypertension symptoms, which is associated with decreased levels of norepinephrine, improved myocardial cell morphology, and reduced inflammation and oxidative stress in the paraventricular nucleus (PVN) (Li et al., 2024). This finding suggests that TGR5 may serve as a central regulator of blood pressure, modulating sympathetic nerve activity and cardiovascular function.

Moreover, the gut microbiota plays a pivotal role in blood pressure regulation, and changes in its composition are closely linked to hypertension (Ge et al., 2024). TGR5 is closely related to gut microbiota function, as evidenced by the significant remodeling of the gut microbiota observed in TGR5^−/−^ mice (Zhan et al., 2024; Ma et al., 2020). This further confirms the role of TGR5 in gut microbiota regulation. Bile acids, as ligands of TGR5, not only activate TGR5 but also regulate the composition and function of the gut microbiota, forming a potential feedback loop for blood pressure regulation (Ishimwe et al., 2022). Table 1 briefly summarizes the role of TGR5 and aging-related CVDs.

**TABLE 1:** Roles of TGR5 in aging-related cardiovascular diseases.

Cardiovascular disease	Role of TGR5	Key evidence	References
*Atherosclerosis*	Reduces Oxidative Stress and Inflammation	TGR5 activation downregulates SR-A and CD36 expression in macrophages, reducing oxLDL uptake and foam cell formation	Sheikh and Salma (2024)
	Improves Lipid Metabolism	TGR5 activation significantly reduces plasma total cholesterol and triglyceride levels, reducing atherosclerotic lesion areas in the aortic root	Queen et al. (2020)
*Myocardial Infarction*	Mitigates Inflammation and Improves Cardiac Function	DCA binding to TGR5 improves cardiac function and reduces ischemic injury in MI mouse models	Hodgson et al. (2020)
	Regulates CD4^+^ T Cell Function	In TGR5^−/−^ mice, elevated CD4^+^ T cell infiltration in ischemic areas leads to more severe ischemic injury and reduced cardiac function post-MI.	Lacanna et al. (2019)
	Enhances Cell Survival and Proliferation	TGR5 activation improves cell proliferation, reduces apoptosis, and decreases inflammatory factor levels in cardiomyocytes subjected to ischemia/reperfusion injury	Ali et al. (2020)
*Heart Failure*	Promotes Myocardial Repair and Regeneration	TGR5 activation likely plays a pivotal role in post-MI cardiac repair and remodeling, preventing HF progression	Lacanna et al. (2019), Jin et al. (2024)
	Reduces Inflammation	DCA binding to TGR5 suppresses the expression and activation of IL-1β, mitigating inflammation-induced myocardial damage	Lun et al. (2024)
*Cardiac Hypertrophy*	Inhibits Hypertrophic Marker Expression	TGR5 activation reduces mRNA levels of hypertrophic markers such as CaN and NFAT in cardiac hypertrophy models	Zhao et al. (2022)
	Modulates Intracellular Calcium Homeostasis	TGR5 activation increases cAMP levels, activating PKA and increasing the phosphorylation of PLN, reversing the downregulation of SERCA2a expression	Zhao et al. (2022)
	Inhibits Fatty Acid Uptake	TGR5 activation inhibits CD36-mediated fatty acid uptake and lipotoxicity, improving energy metabolism and inflammatory responses in cardiac cells	Bhimanwar and Mittal (2022)
*Cardiac Fibrosis*	Inhibits Collagen Fiber Deposition	TGR5 activation reduces the expression of inflammatory factors and the generation of oxidative stress products in myocardial tissue, inhibiting collagen fiber deposition	Wang et al. (2023), Perino et al. (2021)
	Regulates ECM Synthesis and Degradation	TGR5 activation suppresses the activation and proliferation of cardiac fibroblasts, reducing the expression of fibrosis factors such as TGF-β	Tanaka et al. (2024), Holter et al. (2020)
*Hypertension*	Regulates Sodium and Water Balance	TGR5 activation inhibits the expression of ENaC in the kidney, decreasing sodium reabsorption and lowering blood pressure	Gou et al. (2023)
	Promotes Vasodilation	TGR5 activation induces the expression of eNOS and the production of NO, promoting vasodilation and reducing blood pressure	Bidault-Jourdainne et al. (2021), Chiang and Ferrell (2020)
	Modulates Gut Microbiota	TGR5 is closely related to gut microbiota function, and bile acids, as ligands of TGR5, regulate the composition and function of the gut microbiota, affecting blood pressure regulation	Chang et al. (2024), Guo et al. (2024)

## 4 Conclusion and prospect

In summary, this review highlights the emerging role of TGR5 as a potential therapeutic target for aging-related CVDs.By extensively examining the literature, we found that TGR5, a bile acid receptor, plays a crucial role in regulating these mechanisms. These effects are particularly relevant in the context of aging-related CVDs such as atherosclerosis, MI, HF, cardiac hypertrophy, myocardial fibrosis, and hypertension.

Although current evidence suggests that TGR5 activation may confer cardioprotective benefits, the underlying mechanisms remain to be fully elucidated. The current research on TGR5 is limited by several critical gaps. First, the lack of clinical data on safety and efficacy in humans represents a significant barrier to progress. Second, the complexity of TGR5 signaling networks, including its crosstalk with other bile acid receptors such as FXR and PXR, may obscure target-specific effects and complicate therapeutic outcomes. Third, the impact of aging-related microenvironmental changes, including epigenetic modifications and senescence-associated secretory phenotypes, on TGR5 functionality remains poorly understood. Future research should prioritize the development of tissue-selective TGR5 modulators to minimize off-target effects, employ advanced models such as organoids or humanized animals to dissect individual response heterogeneity, and explore potential synergies between TGR5 agonists and existing anti-aging therapies, such as Senolytics, to enhance therapeutic efficacy (Zhang et al., 2022). Additionally, longitudinal studies are needed to assess the long-term safety and tolerability of TGR5-targeted interventions, particularly in elderly populations with comorbidities. Overall, this review underscores the importance of TGR5 in the pathogenesis of aging-related CVDs and highlights its potential as an attractive therapeutic target for these increasingly prevalent conditions.
